# Benefiting from Spontaneously Generated 2D/3D Bulk‐Heterojunctions in Ruddlesden−Popper Perovskite by Incorporation of S‐Bearing Spacer Cation

**DOI:** 10.1002/advs.201900548

**Published:** 2019-05-17

**Authors:** Yajie Yan, Shuang Yu, Alireza Honarfar, Tõnu Pullerits, Kaibo Zheng, Ziqi Liang

**Affiliations:** ^1^ Department of Materials Science Fudan University Shanghai 200433 China; ^2^ Department of Chemical Physics and NanoLund Lund University Box 124 22100 Lund Sweden; ^3^ Department of Chemistry Technical University of Denmark DK‐2800 Kongens Lyngby Denmark

**Keywords:** 2D Ruddlesdden–Popper perovskites, 3D phase, air stability, low‐temperature fabrication, planar solar cells

## Abstract

2D Ruddlesden–Popper (RP) perovskite solar cells have manifested superior operation durability yet inferior charge transport compared to their 3D counterparts. Integrating 3D phases with 2D RP perovskites presents a compromise to maintain respective advantages of both components. Here, the spontaneous generation of 3D phases embedded in 2D perovskite matrix is demonstrated at room temperature via introducing S‐bearing thiophene−2−ethylamine (TEA) as both spacer and stabilizer of inorganic lattices. The resulting 2D/3D bulk heterojunction structures are believed to arise from the compression‐induced epitaxial growth of the 3D phase at the grain boundaries of the 2D phase through the Pb−S interaction. The as‐prepared 2D TEA perovskites exhibit longer exciton diffusion length and extended charge carrier lifetime than the paradigm 2D phenylethylamine (PEA)‐based analogues and hence demonstrate an outstanding power conversion efficiency of 7.20% with significantly increased photocurrent. Dual treatments by NH_4_Cl and dimethyl sulfoxide are further applied to ameliorate the crystallinity and crystal orientation of 2D perovskites. Consequently, TEA‐based devices exhibit a stabilized efficiency over 11% with negligible hysteresis and display excellent ambient stability without encapsulation by preserving 80% efficiency after 270 h storage in air with 60 ± 5% relative humidity at 25 °C.

Organometal halide perovskites have recently attracted significant attention in photovoltaics for its prominent intrinsic characteristics such as low exciton binding energy, high absorption coefficient, and long carrier diffusion length.^1^, ^2^ Despite the unprecedented device performance with the certified record power conversion efficiency (PCE) reaching 24.2%,^3^ the perovskite solar cells (PSCs) suffer from operational instability under ambient moisture^4^, ^5^, ^6^ and oxygen.^7^, ^8^ Various solutions such as encapsulation,^9^, ^10^ interface engineering,^11^, ^12^ and precursor solution chemistry^13^, ^14^ have been developed to reconcile the gap between device performance and stability. Capping the inorganic sheets of Pb−I octahedrons with large hydrophobic organic molecules to form a sandwiched structure, namely, two dimensional (2D) Ruddlesden–Popper (RP) perovskites, has emerged as one of the most promising methods to achieve long‐term durability.^13^, ^14^, ^15^, ^16^, ^17^ Still, the photovoltaic performance of 2D RP perovskites is largely constrained by their intrinsic molecular structures.

The generic molecular formula of 2D RP lead iodide perovskites can be written as (L)_2_A*_n_*_−1_Pb*_n_*I_3_ *_n_*_+1_ where L^+^ is alkylammonium cation—typically phenylethylamine (C_8_H_11_N, PEA) and butylamine (*n*‐BA), A^+^ is monovalent organic cations such as CH_3_NH_3_^+^, and the *n* value is the number of Pb−I octahedral layers between adjacent spacers.^18^ Given the insulating nature of organic spacer (L) and unfavored orientation with (0k0) facets parallel to substrate in 2D RP perovskites, the cross‐plane charge transport between electrodes is severely retarded.^19^ Besides, the bandgap (*E*_g_) of 2D perovskites increases with narrowing the thickness of the quantum wells (i.e., decreasing the *n* value), which is incompatible with making use of the full solar spectrum.^20^ One feasible solution is to introduce three dimensional (3D) perovskite phases into the 2D inorganic [PbI_6_]^4−^ lattices to provide additional transport paths and to lower *E*_g_ for better absorption of the redder part of the solar spectrum. Three major strategies of phase engineering have been demonstrated effective in enhancing device performance, including the construction of 2D/3D bilayer structures, the formation of 2D:3D mixtures, and an increase of *n* values to generate quasi‐3D phases.^21^, ^22^, ^23^, ^24^, ^25^, ^26^, ^27^ For instance, Yang and coworkers constructed 3D/2D (MAPbI_3_/PEA_2_Pb_2_I_4_) bilayer perovskites with improved stability, reduced interface charge recombination and suppressed ion migration, yielding an overall PCE of 19.89%.^22^ The Nazeeruddin group fabricated one‐year stable devices by mixing the 2D (HOOC(CH_2_)_4_NH_3_I:PbI_2_ and 3D CH_3_NH_3_I:PbI_2_ precursors at different molar ratios.^23^ The Etgar team adjusted the *n* values to obtain quasi‐3D perovskites (*n* = 40, 50, and 60), resulting in high open‐circuit voltage (*V*_OC_) and PCE.^24^ All these methods, however, deliberately introduced 3D or quasi‐3D phase into 2D perovskites and hence required either multistep sequential deposition or post‐treatment of thin film (e.g., thermal annealing), which not only adds complexity to device preparation but also reduces the miscibility between 2D and 3D phases and potentially introduces defects at the 2D/3D interface. In addition, the solution processes would preferentially create a vertical cascade of stacked 2D phases with *n* values increasing from bottom (2D phases) to top (quasi‐3D phases) during spin‐casting. Such stacked structures, however, are not favorable for charge collection as the photo‐generated charges from device bottom should all undergo an extra interphase transfer process before reaching the 2D/3D interface to be separated.^28^

In this work, we for the first time demonstrate a spontaneous generation of 2D:3D bulk‐heterojunction structures at room temperature, in which 3D phases are epitaxially connected with 2D lattices by introducing S‐bearing thiophene‐2‐ethylamine (C_6_H_9_NS, TEA) as an organic spacer. X‐ray photoelectron spectroscopy spectra confirm the strong interactions between S and Pb atom, which presumably act to weaken the long‐range order of 2D inorganic lattices and create the nucleation sites for forming 3D phases. Various optical characterization and electron microscopy analysis methods are combined to verify the mixed 2D/3D structures and elucidate the relative spatial organization between them. The 3D‐embedded TEA‐based 2D perovskites exhibit longer exciton diffusion length and extended charge carrier lifetime than the paradigm 2D PEA analogues, which result in more efficient inter‐phase charge carrier transfer and yield a prominent PCE of 7.20% in TEA 2D perovskites based planar PSCs. With dual treatments of NH_4_Cl additives and dimethyl sulfoxide solvent (DMSO), the devices exhibit a remarkably higher PCE up to 11.32% with negligible hysteresis. Moreover, our devices without encapsulation show excellent stability which can sustain 80% photovoltaic performance of post “burn‐in”efficiency after 270 h storage at 25 °C and in air with a relative humidity (RH) of 60 ± 5%.

The 2D RP perovskite (*n* = 4) thin films were made by one‐step spin‐coating of the mixture precursors of PbI_2_, HI, CH_3_NH_3_I, and TEA or PEA in DMF solution at a stoichiometric ratio of 4:2:3:2. The resulting crystal structure of TEA_2_MA_3_Pb_4_I_13_ is schematically displayed in **Figure** **1**a, where four layers of inorganic [PbI_6_]^4−^ octahedrons are sandwiched between organic spacing cations of TEA. Figure 1b compares the X‐ray diffraction (XRD) patterns of TEA_2_MA_3_Pb_4_I_13_ with MAPbI_3_ and PEA_2_MA_3_Pb_4_I_13_ and the corresponding peak parameters are summarized in Table S1 in the Supporting Information, respectively. Both TEA and PEA samples feature the diffraction peaks around 14.10° and 28.50°, which are assigned to the (111) and (220) crystal planes of 2D perovskites, respectively. The locations of 2D characteristic peaks at lower angles—4.06°, 5.61°, 6.51°, 8.00°, and 9.51° for TEA perovskite are assigned to (020) (*n* = 2), (040) (*n* = 4), (020) (*n* = 1), (060) (*n* = 4), and (060) (*n* = 3) facets, respectively; 6.49° and 9.39° for PEA perovskite originate from the (020) (*n* = 1) and (060) (*n* = 3) diffraction planes, respectively. Notably, there is a slight shift to larger diffraction angles from MAPbI_3_ (14.10°), PEA (14.12°) to TEA (14.16°) samples as displayed in the inset of Figure 1b, indicative of noticeable lattice compression in TEA compared to PEA perovskites. The film morphology of both 2D perovskite samples was further evaluated by field‐emission scanning electron microscopy (FE‐SEM) images as shown in Figure 1c,d. Both thin films display similar surface topography with cross‐linked crystals and few pinholes on the surface, implying imperfect crystallization in the absence of optimization.

**Figure 1:**
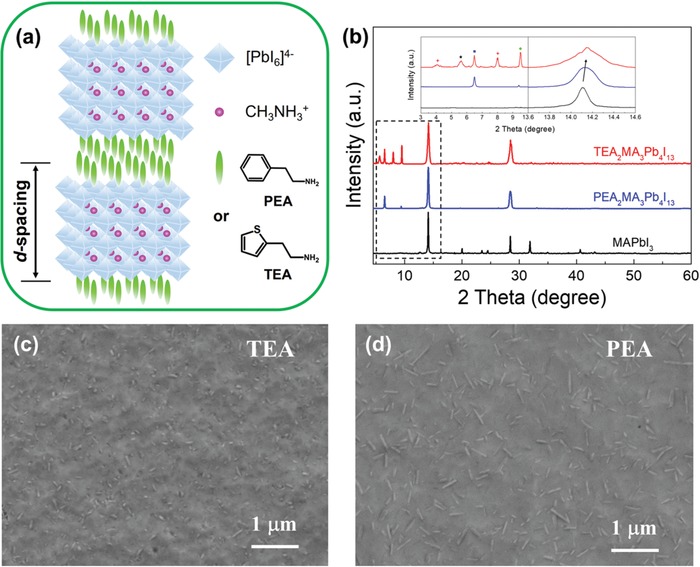
a) Schematic crystal structure of 2D RP perovskites. b) XRD patterns of 3D MAPbI_3_, PEA‐ and TEA‐based 2D perovskite thin films, respectively, with an inset showing magnified diffraction patterns. Top‐view FE‐SEM micrographs of c) TEA and d) PEA samples on glass substrate.

**Figure** **2**a,b presents the optical absorption and photoluminescence (PL) spectra of PEA and TEA thin films, respectively. It is generally believed that solution processed 2D RP perovskite films comprise a mixture of pure 2D phases (*n* ≤ 4), quasi‐2D phases (*n* = 5−20), and neat 3D phases (*n* > 20) with a reduction of *E*_g_ due to weakening of quantum confinement.^18^ For both TEA and PEA, distinctive exciton bands around 515, 570, 610, and 640 nm are observed, which are correlated to 2D quantum wells with *n* = 1−4, respectively^29^ and agree well with low‐angle diffractions in XRD pattern. Apart from 2D phases, the absorption spectrum of TEA perovskite exhibits a distinctive absorption edge around 750 nm which is consistent with the optical *E*_g_ of conventional 3D MAPbI_3_ perovskite. The absorption edge of PEA perovskite films is significantly blue‐shifted compared to TEA as shown in the inset of Figure 2a. This indicates the spontaneous formation of additional 3D phases within TEA 2D perovskites whereas only pure 2D or quasi 2D phases are present in PEA samples with the identical preparation method. Further evidence to the existence of 3D phases is provided by the PL spectra (Figure 2b) where a significant red shift of the emission peak at 760 nm is found in TEA relative to PEA (728 nm). Our previous work established that the interphase charge transfer in 2D perovskites generally follows a trend from small‐*n* phases (2D or quasi‐2D) to large‐*n* ones (quasi‐3D or 3D).^29^ This suggests that the emission peak originates from the radiative recombination of photogenerated charge carriers after an ultrafast interphase transfer to the 3D phase in TEA and the large‐*n* 2D phase in PEA. In addition, the PL emission of TEA film with photo‐excitation from the back‐side is found almost identical to that from front‐side illumination while in PEA films the emission from the 2D phases still remains (black dashed line). This suggests the absence of cascade‐stacking multidimensional phases from large‐*n* to small‐*n* values on the substrate in TEA sample, which is markedly observed in conventional 2D RP perovskite films.^28^ We further measured the Pb and S contents from top (surface) to bottom (interface with the substrate) of TEA samples by depth etching X‐ray photoelectron spectroscopy (XPS) and the obtained atomic ratio of Pb:S is displayed in Figure S1 in the Supporting Information. The nearly constant Pb:S ratio of 2:1, which corresponds to an *n* value of 4 from top surface to bottom interface, confirms the homogeneous phase distribution across the film and indicates the generation of bulk‐heterojunction (BHJ) structures in TEA samples.

**Figure 2:**
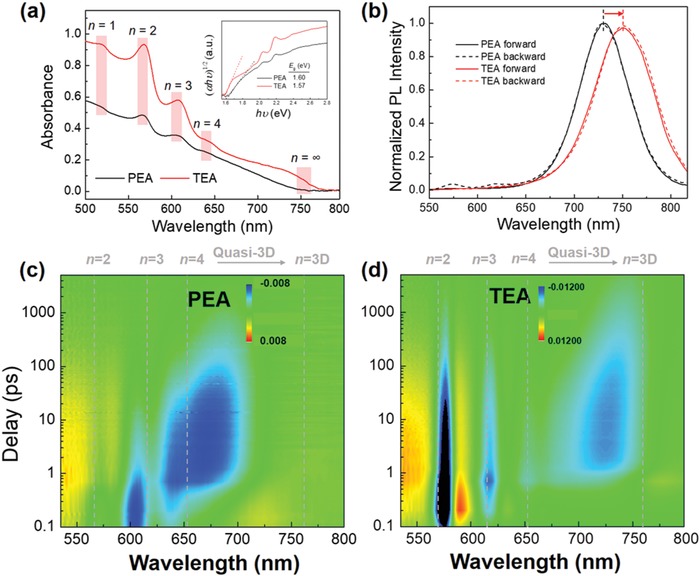
a) Optical absorption and b) PL spectra of neat PEA (black) and TEA (red) thin films, and their TA spectrograms of c) TEA and d) PEA samples. Inset in (a): tauc plots of PEA and TEA films.

The excited state population visualized in transient absorption (TA) spectra further deciphers the phase compositions of PEA and TEA samples as shown in Figure 2c,d, respectively. Both samples were excited with femtosecond laser pulses at 400 nm corresponding to photon energy well above *E*_g_ providing nonselective excitation of all the phases. The initial highly nonequilibrium distribution of charges/excitons will rapidly thermalize populating the lowest states of each phase, which correspond to signature negative bleach bands at different wavelengths in TA spectrograms. These include the bleach of 2D*_n_*_=2_ (560 nm), 2D*_n_*_=3_ (610 nm), 2D*_n_*_=4_ (640 nm), quasi‐2D (650−700 nm), and 3D (750 nm) phases as shown in Figure 2c,d (blue areas). TA spectra of PEA perovskites mainly consist of bleach bands of 2D and quasi‐2D phases while for TEA perovskites, bleach bands at 750 nm clearly reveal the excited state population of 3D phase, which is in good agreement with the absorption spectra. It should be noted that the singular value decomposition (SVD) fitting of the TA spectrograms (Figure S8, Supporting Information) confirms the efficient charge carrier transfer from the 2D phase to 3D phase in TEA which is absent in PEA film.^29^ This is also consistent with the PL spectra results in Figure 2b that only emission from 3D phase can be observed in TEA film. In short, both steady‐state (UV–vis and PL spectra) and transient (TA spectra) optical characterization establish the excitonic band of 3D phases generated within TEA perovskites and such 2D/3D BHJ structures are verified by depth etching XPS profiles.

Scanning transmission electron microscope (STEM) imaging and corresponding energy‐dispersive X‐ray spectroscopy (EDS) mapping were combined to investigate the spatial distribution between the 2D and 3D mixed phases in TEA perovskite as displayed in **Figure** **3**a−c. Considering the homogeneity of TEA perovskite thin film unveiled by depth etching XPS characterization, we exploited the powder sample for imaging with the least damage in the sample preparation (see the details in Experimental Section). Given the sulfur (S) atom in thiophene ring, it is straightforward to distinguish S‐deficient 3D phases from S‐rich 2D domains via EDS mapping. The unevenly dispersed red spots representative of S elements in Figure 3b indicate a mixture of phases with overwhelming 2D (red‐aggregated area) perovskites surrounding 3D (red‐vacant area) ones, which are also observed in lead (Pb) mapping in Figure 3c. Furthermore, in situ TEM imaging was conducted to intuitively confirm the existence of both 2D and 3D phases as shown in Figure S2 in the Supporting Information. There is a clear fingerprint of 2D lattice fringes which are analyzed with fast Fourier transform (FFT) analysis. Interestingly, the FFT analysis shows two sets of diffraction spots with different interplane distances (15.7 and 2.5 Å), which matches well with the reflection of (040) (*n* = 4)^30^ and (320) planes from 2D and 3D perovskites, respectively. Such overlapped diffraction patterns directly verify the mixed phases in TEA perovskites. To offer a deeper insight into the relative position of 2D and 3D perovskite crystals, the selected region of interest was magnified and high‐resolution TEM (HR‐TEM) imaging was performed as shown in Figure 3d−f. On one hand, both region A and B display 2D lattice fringes (yellow) with different orientations, which represent two separate crystals. On the other hand, region C shows densely aligned lattice fringes with an interplanar distance of 3.6 Å, which corresponds to (211) facets of 3D phase. This clearly shows that the extra 3D phase in TEA samples is embedded among the 2D grains, building up BHJ‐like structures, which are distinct from the vertical stacking structures in traditional 2D perovskite thin films.^19^ More importantly, we notice that the lattice transition at the edge between 2D and 3D phases is smooth without interfacial void or defects as shown in Figure 3f, which indicates that the epitaxial growth of 3D phases would probably occur at the grain boundaries of 2D phases.

**Figure 3:**
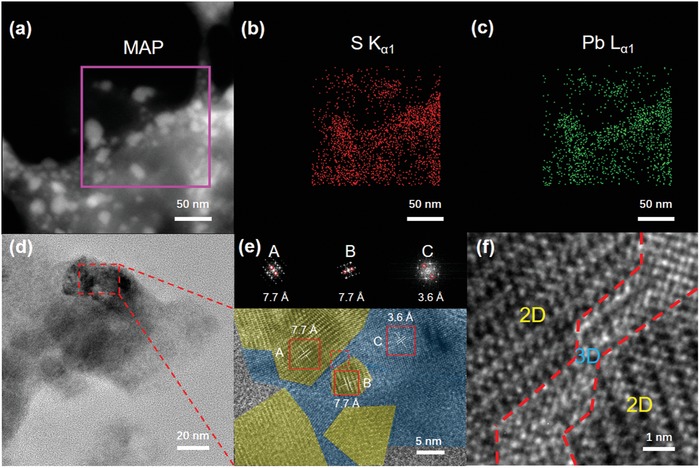
a) STEM images and corresponding EDS elemental mapping showing the distribution of b) sulfur (S) and c) lead (Pb) in TEA perovskites. d) TEM and e) magnified HR‐TEM image of TEA perovskites. Insets in (e) show FFT analysis of the areas within A, B and C boxes, respectively. f) Partial enlargement of the dotted square in (e) which is characteristic of 2D/3D phase transition zone.

Next, we examined the influence of the generated BHJ structures on charge carrier dynamics in TEA perovskites. As mentioned above, the PL emission represents the radiative charge carrier recombination in the final phase after interphase charge transfer. Time‐resolved PL (TRPL) spectroscopy was further utilized to investigate the influence of 3D phases on the charge carrier recombination in TEA 2D perovskites as shown in **Figure** **4**a. The PL decays of both PEA and TEA samples can be best fitted by a biexponential function in Equation (1):

(1) $$I\left(t\right)=A_{1}\exp\left(-\frac{t}{\tau_{1}}\right)+A_{2}\exp\left(-\frac{t}{\tau_{2}}\right)$$

where *A*_1_ and *A*_2_ are the amplitudes, τ_1_ and τ_2_ are the PL decay times. According to our previous studies in 2D perovskites,^31^] τ_1_ corresponds to the PL quenching from fast nonradiative recombination which is induced by defects or impurities introduced during preparation of grains or interphase charge transfer. The slower time constant τ_2_ represents radiative recombination of the charge carriers. There is an obvious increase in τ_2_ from PEA ( 16.4 ns) to TEA ( 19.9 ns), corresponding to the extended charge carrier lifetime of 3D phases in TEA perovskites compared with quasi‐2D phases in PEA perovskites.

**Figure 4:**
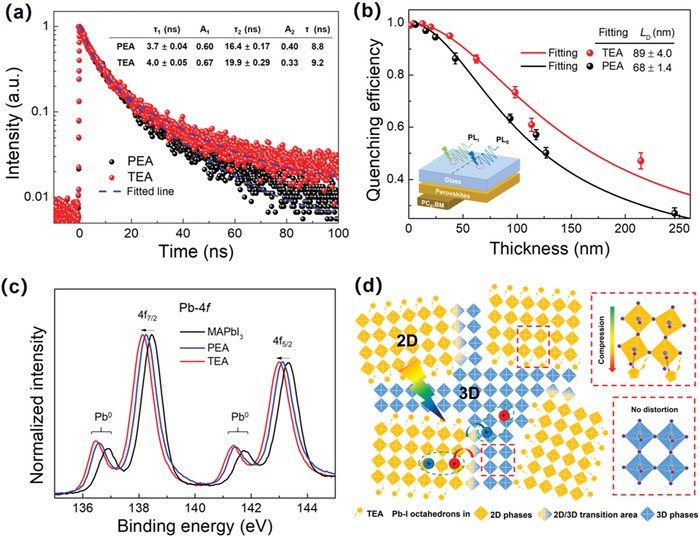
a) TRPL kinetics of PEA‐ and TEA‐based 2D perovskite thin films and the corresponding PL lifetime. b) Determination of *L*_D_ for TEA and PEA neat films. Inset in (b): The schematic diagram of *L*_D_ measurements. c) XPS spectra of 3D MAPbI_3_ perovskite (black), PEA (blue)‐ and TEA (red)‐based 2D perovskite thin films for Pb 4f, respectively. d) Schematic illustration of 2D/3D mixed phases in TEA perovskites.

To gain an in‐depth understanding of the differences in carrier transport in TEA‐ and PEA‐based 2D perovskites, the exciton diffusion length (*L*_D_) was measured as shown in Figure 4b with the inset illustrating the measurement setup. Experimentally, various perovskites thin films with different thicknesses were spin‐casted by varying both the solution concentration and spin rate. The PL spectra were measured from the substrate backside with the corresponding area marked for sequential measurements. Then the PC_61_BM layer, which ensures efficient quenching of emission from photo‐generated excitons near the interface, was deposited onto the as‐casted perovskite thin films. Afterward, the PL measurements were performed again at the same location as in the first step to generate the quenched PL spectra. It was reported that the ratio of quenched/unquenched PL emission–quenching efficiency (*Q*_e_) decreases as a function of thickness of active layer.^32^ *L*_D_ was thus extracted from the fitted curve of *Q*_e_ versus the thickness of the perovskite films using the Equation (2), 32

(2) $$Q_{e}=1-\frac{PL_{Q}}{PL_{i}}=\;\frac{L_{D}}{d}\times tanh\left(\frac{d}{L_{D}}\right)$$

where *Q*_e_ is the relative decrease percent of PL intensity caused by quench layer, *PL*_i_ and *PL*_Q_ are the integrated PL intensity before and after quench treatment, *L*_D_ is the diffusion length, *d* is the thickness of the perovskite films. *L*_D_ is determined to be 89 ± 4.0 nm in TEA in comparison with 68 ± 1.4 nm in PEA. It should be noted that the *L*_D_ has contributions from both the diffusion within 2D phases and the transport within 3D phases. We argue that the dissociation of excitons already occurred at the boundary of 2D phases as also seen in another report.^29^ The longer lifetime and larger diffusion length in TEA reveals the enhanced charge carrier transport in the BHJ structures. It also indicates a more efficient interphase charge transfer process in TEA compared with the PEA samples, which is supported by the detailed analysis of TA results shown in Figure 2c,d and Figure S8 in the Supporting Information where the transfer rates are extracted from SVD fitting of the TA spectrograms. Besides, the TA results clearly show that the charge carrier lifetime in 3D phases is about 20−30 ns, which is comparable to that of 3D MAPbI_3_ film. Since it is widely accepted in MAPbI_3_ that the trap tolerance and dielectric screening can facilitate charge transportation and reduce charge recombination rates, it should be still valid in the 3D phases of our samples.^33^, ^34^

The superior optoelectronic and charge carrier transport properties of TEA‐based 2D perovskites compared to the PEA analogues is most likely correlated to the embedded 3D phases. However, the 3D phases formed remain elusive. Since S atom is believed to easily bond with Pb atom, we suggest that S‐bearing thiophene rings will interact with Pb−I octahedrons, form strong dipoles with Pb atoms, and result in large distortions in 2D lattices, which directly modulates the crystallization dynamics and induces the formation of 3D phases. Thus, we employed XPS to elucidate the interaction between Pb−I octahedrons and the S‐contained organic spacer as displayed in Figure 4c (see Figure S3a in the Supporting Information for detailed peak fitting data). All of the XPS spectra were calibrated by using C 1s spectra. It is well known that the binding energy (*E*_B_) depends on the local chemical environment. We found that the characteristic peaks of both Pb 4f_7/2_ and Pb 4f_5/2_ shift toward low *E*_B_ with an increasing trend of TEA (138.13 and 143.01 eV) < PEA (138.24 and 143.11 eV) < MAPbI_3_ (138.46 and 143.31 eV) as shown in Figure 4c. As the intrinsic oxidation states are the same in these three samples, such a red shift should be attributed to the increased electron cloud overlap between Pb and halide atoms in the lattices.^35^ One of the reasons could be the lattice compression induced by the long‐chain spacer cations in 2D perovskites, which is clearly verified in XRD measurement (Figure 1b) where the diffraction peaks are shifted to high angles with corresponding reduced d‐spacing from MAPbI_3_ to PEA and then TEA. Such a lattice compression has also been observed in other 2D perovskite materials.^15^ In addition, the more compressive strain from the TEA cations compared to PEA suggests a strong interaction between Pb and S atoms, which intensifies the coordination between Pb and I atoms. Moreover, the S 2p_3/2_ and 2p_1/2_ bands shift from 164.1 eV (in neat thiophene ring^36^) to 164.4 eV (in TEA based 2D perovskites) (Figure S3b, Supporting Information), which indicates the diluted electron cloud density and hence the weakened shielding effect on S atoms and also verifies the existence of the Pb−S interaction. Besides, the shift of (111) peak to higher 2θ, i.e., decreased d‐spacing in TEA relative to PEA, evidences the lattice compression caused by the Pb−S interaction. Note that the small peaks around 137 and 142 eV are often observed in Pb 4f spectra, which is most likely attributed to the signal of Pb^0^ owning to the X‐ray beam damage during the measurement.^37^, ^38^ These XPS results provide solid evidence for the interaction between thiophene ring and Pb−I octahedrons, which lead to the following merits. First, it improves the electronic coupling between the perovskite octahedrons and the spacing cations at the interface, which not only provides additional driving force for both exciton dissociation and interphase charge transfer as discussed above, but also enhances the stability of the inorganic perovskite lattices compared to PEA‐based perovskites, which is demonstrated from the stability test of thin films at room temperature with a high RH of 70 ± 5 % as shown in Figure S4 in the Supporting Information. Second, it could also explain the origin of simultaneously generated 3D phases in 2D RP perovskite matrix since the increased compressive strain would reduce the long‐range order of the 2D RP lattices which enables the nucleation of the 3D clusters. To further comprehend the charge separation and recombination mechanisms in BHJ structures, the resultant 2D/3D mixed phases are schematically shown in Figure 4d. In PEA‐based devices, most of the photogenerated excitons will recombine rapidly because of the poor charge transportation within 2D phases, while only those in quasi‐2D phases live long enough to be collected. In TEA‐based devices, in contrast, the photogenerated excitons will dissociate under a driving force of the energy band differences. The free electrons and holes will be transferred to the 3D and 2D phases, respectively, and then transported with much lower recombination rates to the electrodes for higher charge collection efficiency.

The above investigations have revealed the origins of mixed 2D/3D BHJ phases in TEA‐based 2D perovskites and their influences on the intrinsic photophysical properties, demonstrating their outstanding potential for PV applications. As a proof‐of‐concept, we fabricated PEA‐ and TEA‐based 2D RP PSCs with an architecture of ITO/PEDOT:PSS/2D perovskites/PCBM/Bphen/Al and the cross‐section SEM imaging of device is shown in **Figure** **5**a. The representative current density−voltage (*J*−*V*) curves under reverse scan and standard AM 1.5G illumination are shown in Figure 5b, and the photovoltaic data are summarized in **Table** **1**. Compared to the PEA counterparts, the TEA‐based devices show a 40% and 35% increase in short‐circuit current density (*J*_SC_) for reverse and forward scans and yield a PCE of 7.20%, which is among the highest ones in low‐temperature fabricated 2D RP PSCs.^13^, ^14^ We believe that such an enhancement greatly benefits from the large‐scale defect‐free 2D/3D interfaces in the spontaneously formed 2D/3D BHJs which ensure efficient exciton splitting. Nevertheless, neat 2D layered perovskite thin films without further modifications present the inherent structural disorder^39^ and orientation randomness,^39^ both of which not only lead to trap‐assisted nonradiative recombination but also restrict charge transport perpendicular to substrate, thus causing serious hysteresis (see Figure S10a in the Supporting Information for details) and limited device performance. Our previous work demonstrated that the crystallinity and crystal orientation in room‐temperature fabricated 2D RP perovskites can be remarkably improved by the dual treatments of NH_4_Cl and DMSO. Specifically, NH_4_Cl functions to promote the crystallization and reduce trap/defect states yet weaken the lattice order of perovskites whereas DMSO acts to retard the crystallization yet improve the vertical orientation of perovskites and optimizes the 2D/3D interfaces, thereby facilitating interphase charge transfer. These two competitive processes achieve an optimal balance in co‐treated device and result in the best device performance.^31^, ^41^ Therefore, we attempted to exploit the same strategy to further optimize the TEA thin films and the detailed verification regarding the improvement of crystal structure and orientation, film morphology as well as charge carrier dynamics by NH_4_Cl and DMSO treatments can be found in Figures S5−S9 and Table S2 in the Supporting Information. The optimal TEA + NH_4_Cl + DMSO (denoted as TND) thin film‐based devices were built on the same device structure as described above and a significant enhancement is achieved, which yields the best PCE of 11.32% with little hysteresis (see Figure S10d and Table S3 in the Supporting Information for details). It is worth noting that after the addition of NH_4_Cl, _V__OC_ increases from 1.02/1.06 V to 1.06/1.08 V for reverse and forward scans, respectively (Figure S10). On the other hand, when treated with DMSO, _V__OC_ decreases down to 0.92/0.92 V, which is similar to that of TEA + NH_4_Cl + DMSO based devices. It seems that NH_4_Cl and DMSO play the opposite roles in influencing _V__OC_. However, such a trend is not applicable to PEA as seen in Table 1. Given the similar molecular structures of PEA and TEA spacers, we speculate that the difference lies in the competing effect between the interaction of TEA and DMSO with Pb−I octahedron, respectively. The XPS results (Figure 4) provide a solid evidence for the interaction between thiophene ring and Pb−I octahedron. Meanwhile, it is widely acknowledged that DMSO (Lewis acid) tends to form intermediates with Pb−I octahedron (Lewis acid) during crystallization. These two processes may compete with each other and finally lower _V__OC_ in DMSO‐treated devices. The Gaussian distribution of PCEs for 69 devices is statistically shown in Figure S11 in the Supporting Information, which exhibits a maximal PCE of 12.54% and an average PCE of 10.03%. We then measured the stabilized PCE over light illumination time by fixing the voltage at the maximum power point of 0.70 V (Figure 5c). The power output remains steady for over 1000 s with a stabilized PCE of 11.18% for TND‐based PSCs. Besides, Figure 5d shows the external quantum efficiency (EQE) spectra of the optimal TND‐based devices. Notably, the integrated *J*_SC_ is 17.84 mA cm^−2^ with a disparity of 6.1% from that obtained from *J*−*V* characteristics, which arises from different responses of the highly oriented TND perovskites to nonpolarized light from the solar simulator and partial polarized light from EQE. Moreover, we carried out device stability test of TND‐based PSCs under various conditions as shown in Figure 5e and Figure S12 in the Supporting Information. Both the TND, PND, and control devices are stored and tested without encapsulation in air atmosphere (25 °C, 60 ± 5% RH). In the humidity stability test, the post “burn‐in” section of the normalized PCE is linearly fitted and then extrapolated back to the *y*‐axis to obtain the intercept (T_0_) and T_80_ (time required for 20% degradation of the T_0_), which are determined to be 270, 207, and 2 h for the TND, PND and control devices, respectively. To examine the device photostability, the maximum power point (MPP) tracking under continuous white light illumination^42^ is exploited as shown in Figure S12 in the Supporting Information and the estimated T_s80_ (corrected T_80_) for TND and PND are determined to be 172 and 75 h, respectively, which agree with the results in the humidity test.

**Figure 5:**
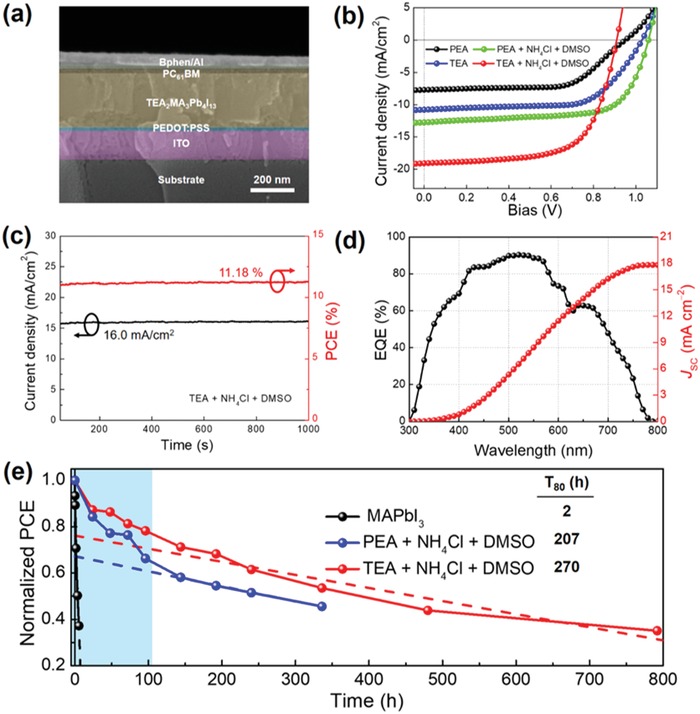
a) Cross‐sectional SEM image of the as‐fabricated device. b) Representative current density−voltage (*J*−*V*) characteristics of PEA‐ and TEA‐based planar PSCs under a light irradiation of 100 mW cm^−2^ at reverse scan. c) Stabilized photocurrent density (black) and PCE (red) of TND‐based PSCs measured under a constant bias of 0.70 V at the maximum power point. d) EQE and integrated *J*_SC_ profiles of the corresponding optimal devices. e) Stability test of the MAPbI_3_, PND‐ and TND‐based PSCs storing at room temperature and a RH of 60 ± 5%.

**Table 1:** Photovoltaic performance parameters of PEA_2_MA_3_Pb_4_I_13_‐ and TEA_2_MA_3_Pb_4_I_13_‐based planar cells

Sample	Scanning direction	*J*_SC_ [mA cm^−2^]	*V*_OC_ [V]	FF [%]	PCE [%]
PEA	Reverse	7.70	0.94	0.65	4.69
	Forward	7.93	1.00	0.53	4.23
TEA	Reverse	10.78	1.02	0.66	7.22
	Forward	10.72	1.06	0.55	6.20
PEA + NH_4_Cl + DMSO	Reverse	12.73	1.06	0.68	9.19
	Forward	12.67	1.06	0.68	9.19
TEA + NH_4_Cl + DMSO	Reverse	19.01	0.90	0.66	11.32
	Forward	18.99	0.92	0.60	11.28

The excellent ambient stability of TND devices results not only from the protection by the hydrophobic organic spacer, but also from the Pb−S interaction that stabilizes the inorganic lattices within perovskites as discussed in Figure 4c. Finally, we compare the key parameters of both device stability and photovoltaic performance between our TND device and the literature reported 2D RP PSCs with similar *n* values as summarized in Tables S4 and S5 in the Supporting Information, respectively. It can be seen that TEA perovskite‐based devices demonstrated in this work situate among the best‐performing PSCs^43^ with an active layer of 2D RP perovskite (*n* = 4 or 5), annealing‐free fabrication and storage conditions (25 °C, 60 ± 5% RH).

In conclusion, we have demonstrated a spontaneous generation of 3D perovskite phase embedded in 2D RP perovskite matrix at room temperature by exploiting S‐contained TEA as the organic spacer. The strong interaction between Pb and S not only stabilizes the perovskite crystal structures but also compresses the perovskite lattices, which induces the nucleation and growth of 3D phases at grain boundaries of 2D perovskite domains. The formed bulk heterojunction structures feature both longer *L*_D_ (89 ± 4.0 nm) and charge carrier lifetime (9.2 ns) than the PEA analogue (68 ± 1.4 nm and 8.8 ns), which explains the remarkably enhanced *J*_SC_ and higher PCE of 7.20% in TEA compared to the PEA‐based PSCs. To further modulate the crystallinity and crystal orientation in TEA based perovskites, NH_4_Cl and DMSO treatments were combined to produce PSCs with both a high stabilized PCE of 11.32% and negligible hysteresis. Besides, the TEA‐based PSCs show a T_80_ of 270 h in ambient atmosphere (25 °C, 60 ± 5% RH) without encapsulation. This work opens up a new avenue to all‐low‐temperature fabricated 2D RP perovskite solar cells that greatly benefit from 2D‐stablized/3D‐transported BHJs.

## Conflict of Interest

The authors declare no conflict of interest.

## Supporting information

Supplementary
