# Supplementary material for: Benefiting from Spontaneously Generated 2D/3D Bulk‐Heterojunctions in Ruddlesden−Popper Perovskite by Incorporation of S‐Bearing Spacer Cation
Source: Adv Sci (Weinh). 2019 May 17;6(14):1900548. doi: 10.1002/advs.201900548 (PMC6661945; doi:10.1002/advs.201900548)
Supplement: Supplementary file 1 — Supplementary [file ADVS-6-1900548-s001.pdf]

Copyright WILEY-VCH Verlag GmbH & Co. KGaA, 69469 Weinheim, Germany, 2019.

ADVANCED SCIENCE
Open Access

Supporting Information

for *Adv. Sci.,* DOI: 10.1002/advs.201900548

Benefiting from Spontaneously Generated 2D/3D Bulk-Heterojunctions in Ruddlesden−Popper Perovskite by Incorporation of S-Bearing Spacer Cation

Yajie Yan, Shuang Yu, Alireza Honarfar, Tõnu Pullerits, Kaibo Zheng, and Ziqi Liang**

Supporting Information

Benefiting from Spontaneously Generated 2D/3D Bulk-Heterojunctions in Ruddlesden−Popper Perovskite by Incorporation of *S*-Bearing Spacer Cation

Yajie Yan[1], Shuang Yu[1], Alireza Honarfar,[3] Tõnu Pullerits[3], Kaibo Zheng[2,3], and Ziqi Liang[1]**

[*][1]Prof. Z. Liang, Y. Yan, S. Yu

Department of Materials Science

Fudan University

Shanghai 200433, China

Email: zqliang@fudan.edu.cn

[*][2]Dr. K. Zheng

Department of Chemistry

Technical University of Denmark

DK-2800 Kongens Lyngby, Denmark

Email: kzheng@kemi.dtu.dk

[3]Dr. K. Zheng, A. Honarfar, Prof. T. Pullerits

Department of Chemical Physics and NanoLund

Lund University

Box 124, 22100 Lund, Sweden

Experimental Section

Materials: PbI_2 and CH_3NH_3I were purchased from Maituowei Ltd. (China). Both thiophene-2-ethylamine and phenethylamine were purchased from Energy Chemical. All other chemicals were purchased from J&K Scientific, Ltd. (China). All the reagents were used as received.

Fabrication of 2D Perovskite Films: The pristine $(TEA)_2MA_3Pb_4I_{13}$ ($(PEA)_2MA_3Pb_4I_{13}$) precursor solutions (40 wt%) were prepared by mixing 0.412 g (0.413 g) PbI_2 (99.999%), 0.100 g (0.102 g) HI (57 wt% aqueous solution), 0.106 g (0.107 g) CH_3NH_3I (99.999%) and 0.057 g TEA (0.054 g PEA) at a stoichiometric ratio of 4:2:3:2 in DMF. For the preparation of the precursors of TEA + NH_4Cl, TEA + DMSO and TEA + NH_4Cl + DMSO samples, additional 7 mg of NH_4Cl, 50 µL of DMSO and both of them were added to the pristine precursor (1 mL), correspondingly. All the 2D perovskites films were obtained by spin-coating the precursor solutions at 5000 rpm for 30 s at room temperature.

Characterization: Optical absorption spectra of samples were acquired on Agilent 8453 UV-Visible spectrophotometer. Steady-state photoluminescence was measured using a FluoroMax@-4 spectrofluorometer (HORIBA JOBIN YVON, Inc., Edison, NJ) with the excitation beam at 500 nm. The PL intensity was then corrected by absorbed photon numbers at the exciting light wavelength. X-ray diffraction pattern data for 2θ values were collected with a Bruker AX D8 Advance diffractometer with nickel filtered Cu Kα radiation (λ = 1.5406 Å). Field-emission scanning electron microscopy images coupled with energy-dispersive X-ray elemental analysis were acquired on Philips XL-30 field-emission gun at an accelerating voltage of up to 30 kV. Grazing incidence wide-angle X-ray scattering (GIWAXS) experiments were carried out at the Shanghai Synchrotron Radiation Facility (SSRF). Samples were prepared on Si substrates using the same preparation conditions as for devices. The data were obtained with an area CCD detector of 3072 by 3072 pixels resolution (225 mm by 225 mm) at beamline BL14B1. The monochromated energy of the X-ray source was 10 keV. The X-ray wavelength was 1.2378 Å and the incidence angle was 0.15°. Transient absorption (TA) experiments

were performed by using a femtosecond pump-probe setup in nitrogen atmosphere. Laser pulses (800 nm, 80 fs pulse length, 1 kHz repetition rate) were generated by a regenerative amplifier (Spitfire XP Pro) seeded by a femtosecond oscillator (Mai Tai SP, both Spectra Physics). The pump pulses at 400 nm were generated by a BBO crystal as a second harmonic of the laser. The used excitation photon fluxes are 3×10^{12} and 1.5×10^{14} photons/cm^2/pulse. For the probe, we used the super-continuum generation from a thin CaF_2 plate. The mutual polarization between pump and probe beams was set to the magic angle (54.7°) by placing a Berek compensator in the pump beam. The probe pulse and the reference pulse were dispersed in a spectrograph and detected by a diode array (Pascher Instruments). In order to avoid photo-damage, the sample was moved to a fresh spot after each time delay point. Global SVD analysis was performed with the Glotaran software package (http://glotaran.org). These methods yield more accurate fits of rate constants because they treat the full data set as a whole. A simple sequential decay model with various components is chosen for every fitting. Time-resolved photoluminescence (TRPL) spectra were obtained using a streak camera (Hamamatsu, C6860). The laser source is an amplified titanium/sapphire laser providing 800 nm 35-fs pulses at 2 kHz which is then frequency doubled for 400 nm excitation. The absolute PL quantum yield (PLQY) was measured by using a standard spectrometer with integrating sphere (Horiba) using the same fs pulse laser as in TA and TRPL measurement. X-ray photoelectron spectroscopy (XPS) spectra were acquired on a ESCALAB 250 spectrometer with a source gun type of Al Kα and an energy step of 0.05 eV. The experiment process is schematically shown in in Figure S1. Firstly, we cleaned the sample surface with Ar^+ sputtering and performed XPS analysis. The content percents of Pb, S, C, N and I were obtained by integrating the corresponding elemental peak(s) versus binding energy and then normalizing the intensity to unity. Transmission electron microscopic (TEM) imaging was performed on JEM-2100 (JEOL Ltd.) at an accelerating voltage of 200 kV. The energy-dispersive X-ray spectroscopy (EDS) were measured on X-Max^N (OXFORD INSTRUMENTS). The sample was made

by scratching the perovskite thin film off from the substrate and sonicating it in toluene for 10 min, followed by dropping the suspension onto an ultra-thin carbon support membrane.

Device Fabrication and Measurements: ITO substrates were cleaned sequentially in an ultrasonic bath with deionized water, acetone, and isopropyl alcohol for 20 min, respectively, and then dried under nitrogen. The substrates were oxidized in UV-ozone for 20 min before use. The PEDOT:PSS layers were spin-coated on the patterned substrates at 3000 rpm for 60 s and annealed at 130 °C for 30 min. The substrates coated PEDOT:PSS were transferred to a N_2 filled glovebox for making the active layers. After the formation of the perovskite layer, a solution of PCBM in chlorobenzene was spin-coated at 3000 rpm for 50 s. Finally, Bphen and Al electrode of 3 nm and 70 nm were thermally deposited with a rate of 0.5 Å/s and 1 Å/s, respectively. The active area as defined shadow mask is ~0.04 cm^2. The sample was mounted inside a nitrogen-filled sample holder with a quartz optical window for subsequent measurements. The light *J–V* curves were measured on a Keithley 2400 source meter unit under AM 1.5G light illumination with a Newport-Oriel (Sol3A Class AAA Solar Simulator, 94043A) solar simulator operating at an intensity of 100 mW cm^{-2}. The light intensity was calibrated by a certified Oriel reference cell (91150V) and verified with a NREL calibrated, filtered silicon diode (Hamamatsu, S1787-04). The *J–V* profiles were obtained under both forward (−0.5 V → +1.5 V) and reverse (+1.5 V → −0.5 V) scans. External quantum efficiency (EQE) spectra were measured on a commercial EQE set-up (QE-R, Enli Technology Co., Ltd). A calibrated silicon diode with a known spectral response was used as a reference.

Results

Table S1. XRD parameters of $MAPbI_3$, PEA and TEA based 2D perovskites

Sample	2-Theta (°)	D (Å)
$MAPbI_3$	14.102	6.2729
	28.449	3.1336
PEA	14.123	6.2668
	28.469	3.1315
TEA	14.163	6.2481
	28.471	3.1312

Table S2. Analysis of biexponetial fit parameters for TRPL kinetics data of TEA-perovskites with different treatment.

Sample	τ_1 (ns)	A_1	τ_2 (ns)	A_2	Average τ (ns)
TEA	4.7	0.73	22.9	0.27	9.6
TEA + NH_4Cl	2.1	0.52	25.4	0.48	13.3
TEA + DMSO	1.7	0.32	35.2	0.68	24.5
TEA + NH_4Cl + DMSO	1.3	0.39	22.4	0.61	14.2

Table S3. Hysteresis Index of PEA, TEA and TND based perovskite solar cells

Sample	Scan rate (mV/s)	Hysteresis index
PEA	20	0.112
TEA	20	0.171

TEA + NH_4Cl + DMSO	20	0.0311

Note: Hysteresis Index = $\frac{J_{RS}(0.8V_{OC})-J_{FS}(0.8V_{OC})}{J_{RS}(0.8V_{OC})}$ where J_{RS} (0.8 V_{OC}) and J_{FS} (0.8 V_{OC}) represent photocurrent density at 80% of V_{OC} for the reverse and forward scan, respectively.

Refer to: Kim, H.-S. & Park N.-G. Parameters affecting I–V hysteresis of $CH_3NH_3PbI_3$ perovskite solar cells: effects of perovskite crystal size and mesoporous TiO_2 layer. *J. Phys. Chem. Lett.* **5**, 2927−2934 (2014).

Table S4. Comparison of device stability between TND and reported 2D perovskite-based PSCs

Active layer	Device structure	Storage conditions	T_{80} (h)	Ref
$(PEA_2PbI_4)_{0.016}(FAPbI_3)_{0.984}$	n-i-p	50% RH, 40 °C, encapsulated	~500	[15]
$MAPbI_3/PEA_2PbI_4$ bilayer	p-i-n	20–30% RH, RT, ambient	~140	[22]
$BA_{0.09}(FA_{0.83}Cs_{0.17})_{0.91}Pb(I_{0.6}Br_{0.4})_3$	n-i-p	45% RH, RT, ambient	1005	[25]
$BA_2MA_3Pb_4I_{13}$	n-i-p	65% RH, RT	36	[44]
$BA_2(MA, FA)_3Pb_4I_{13}$	p-i-n	~40−60% RH, RT, ambient	~1300	[46]
$PEA_2MA_4Pb_5I_{16}$	p-i-n	55 ± 5% RH, RT, ambient	160	[47]
$(PEA)_2(MA)_4Pb_5I_{16}$	p-i-n	~30% RH, RT, encapsulated	>1080	[48]
$TEA_2MA_3Pb_4I_{13}$	p-i-n	60 ± 5% RH, RT, ambient	270	This work

Table S5. Comparison of device performance between TND and reported 2D perovskite-based PSCs

Active layer	Device structure	Treatments	PCE (%)	Ref
$BA_2MA_4Pb_5I_{16}$	p-i-n	Hot-casting (110 °C 10–15 min)	8.71	[30]
$BA_2MA_3Pb_4I_{13}$	n-i-p	5% Cs-doping,<br>TA at 100 °C, 10 min	13.70	[44]
$BA_2MA_3Pb_4I_{13}$	n-i-p	Precursor solution (90 °C)<br>TA at 100 °C, 10 min	12.17	[45]
$BA_2(MA, FA)_3Pb_4I_{13}$	p-i-n	TA at 70 °C, 20 min	12.81	[46]
$PEA_2MA_4Pb_5I_{16}$	p-i-n	NH_4SCN additive<br>TA at 100 °C, 15 min	11.01	[47]
$(PEA)_2(MA)_4Pb_5I_{16}$	p-i-n	NH_4SCN and NH_4Cl additives<br>TA at 100 °C, 10 min	14.10	[48]
$TEA_2MA_3Pb_4I_{13}$	p-i-n	NH_4Cl & DMSO additives<br>RT	12.54 (11.32)	This work

Note: TA = thermal annealing, RT = room temperature.

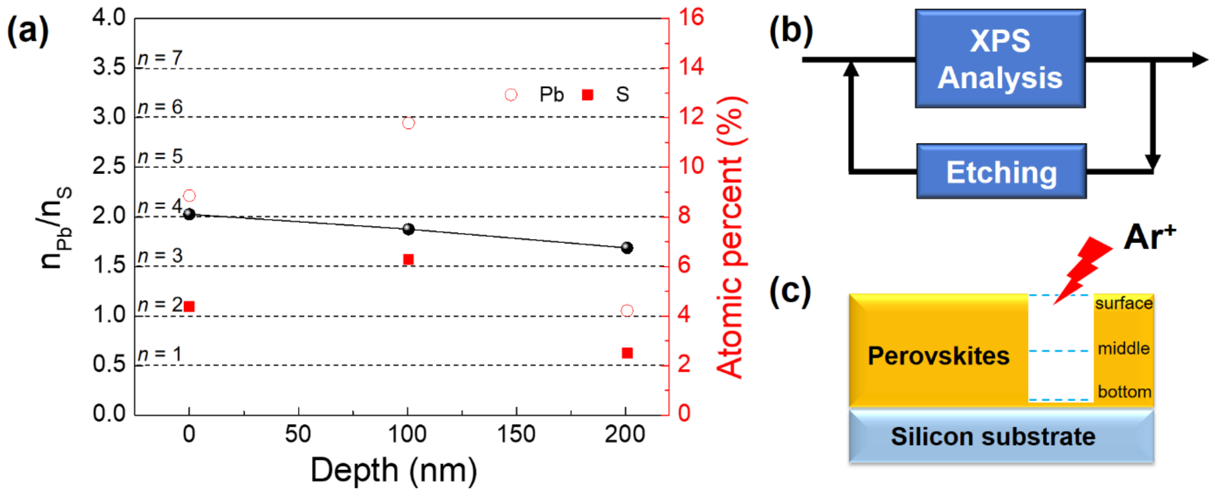

Figure S1. (a) Atomic ratio for Pb and S at different depths of TEA based 2D perovskite thin films. (b) The flow chart and (c) scheme of XPS method to determine the relative content of Pb and S at different depths of samples.

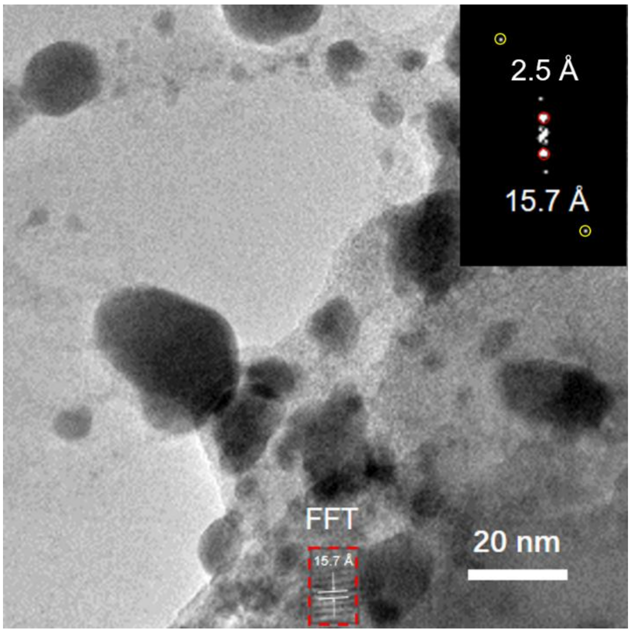

Figure S2. In-situ TEM image of EDX mapping area for TEA based perovskites.

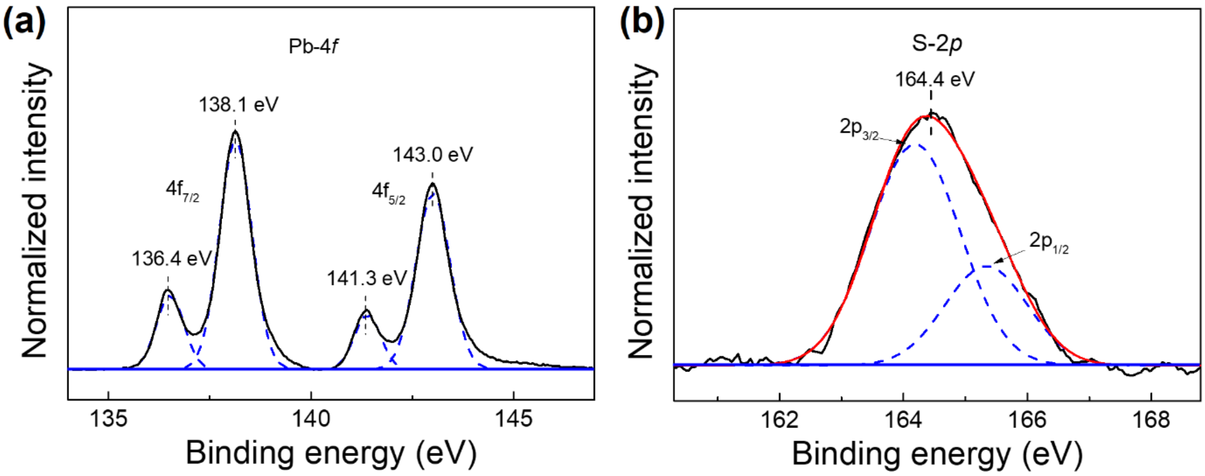

Figure S3. XPS peak fitting results of TEA based 2D perovskite thin films for (a) Pb-4f and (b) S-2p.

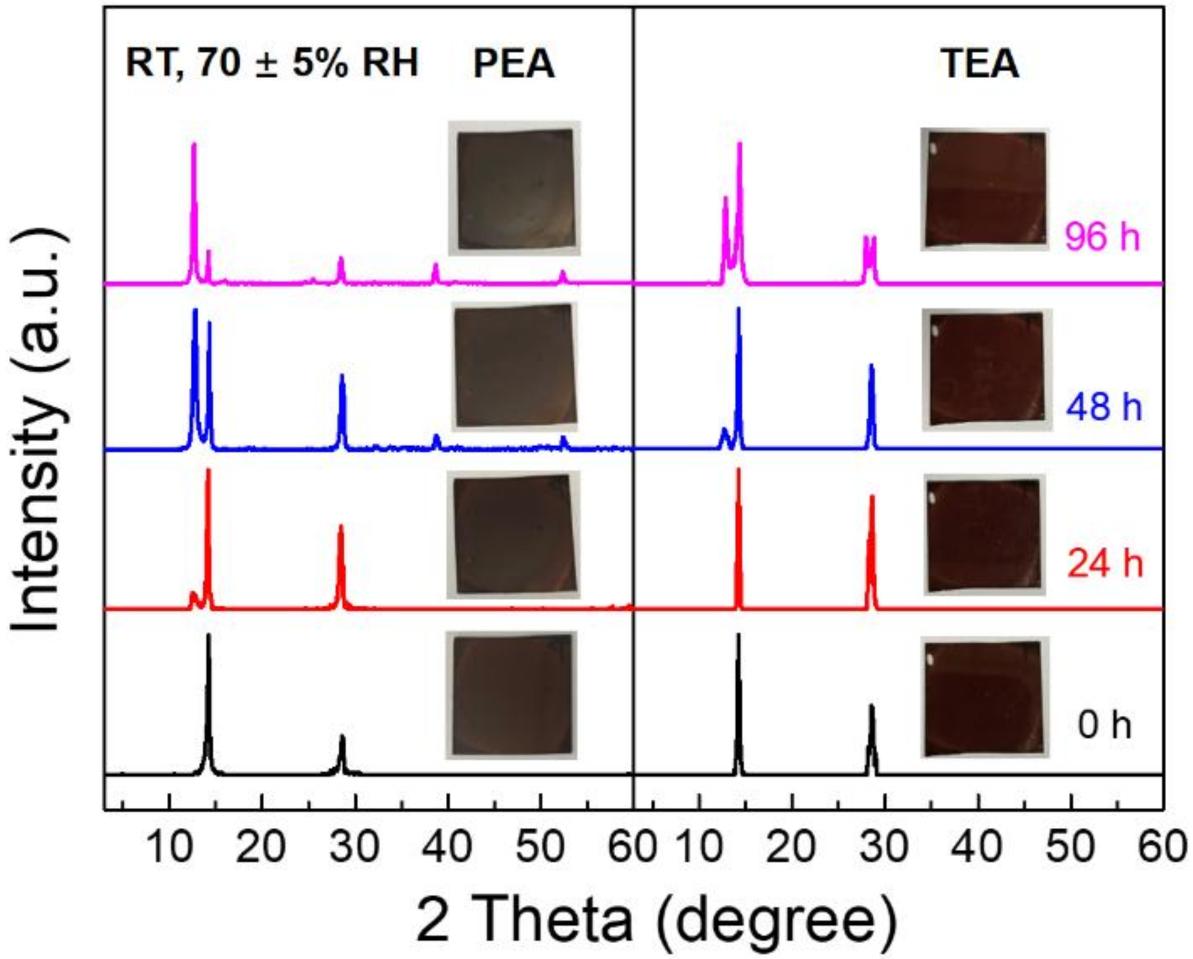

Figure S4. Long-term lattice and film stability comparison between neat PEA and TEA based perovskite thin films at room temperature and a RH of 70 ± 5%.

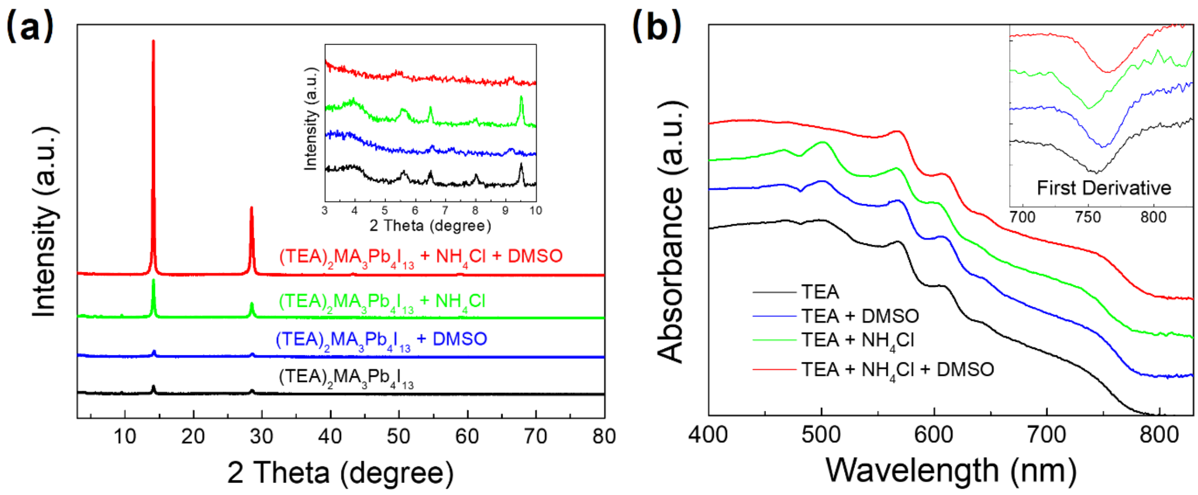

Figure S5. (a) XRD patterns and (b) optical absorption properties of neat TEA and NH_4Cl/DMSO treated samples. Inset in (a): Magnified diffraction patterns in the range of 3−10°, inset in (b): first derivative of the absorbance around 750 nm.

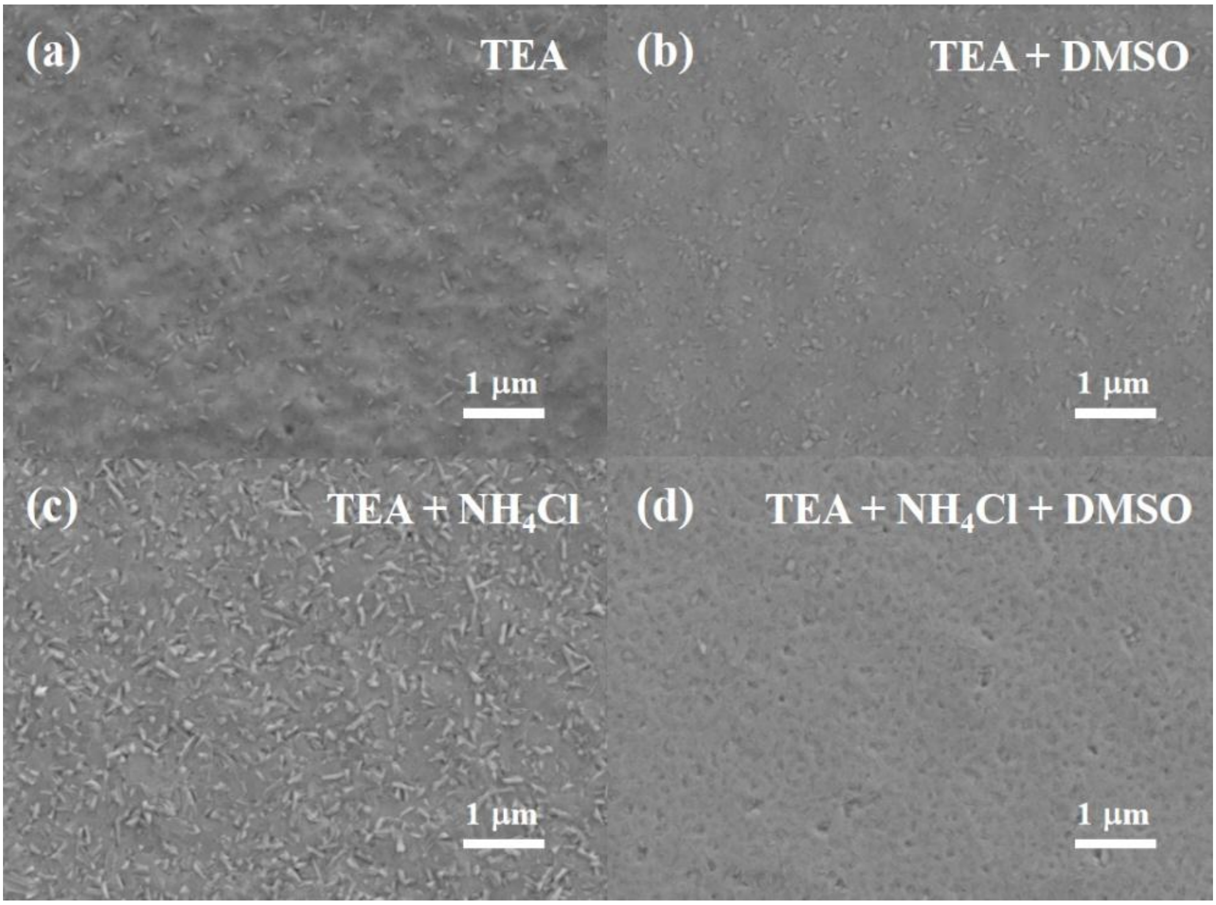

Figure S6. Top-view FE-SEM micrographs of (a) TEA, (b) TEA + DMSO, (c) TEA + NH_4Cl and (d) TEA + NH_4Cl + DMSO. Scale bar = 1 μm.

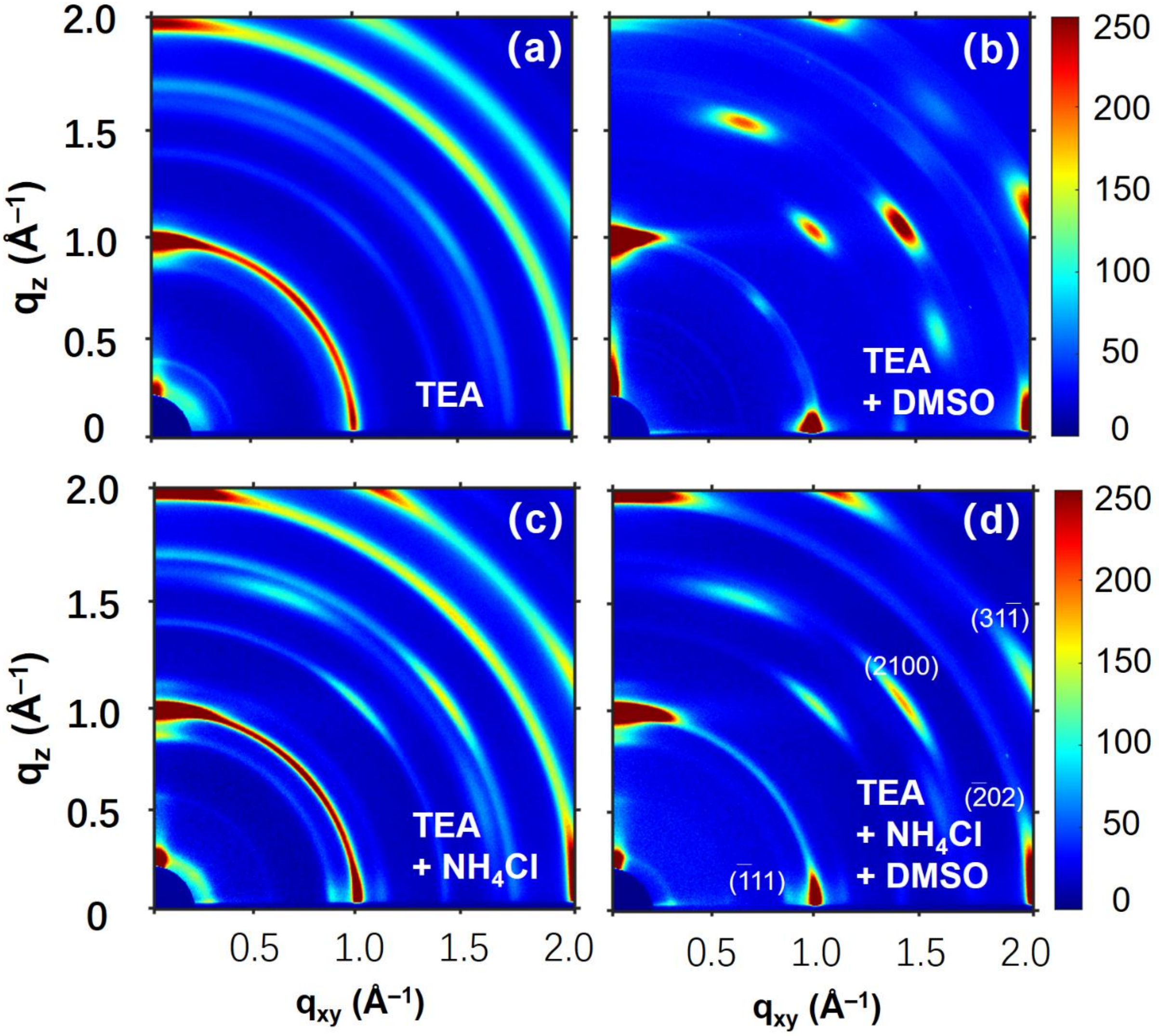

Figure S7. GIWAXS profiles of (a) TEA, (b) TEA+ DMSO, (c) TEA + NH_4Cl, and (d) TEA + NH_4Cl + DMSO, respectively.

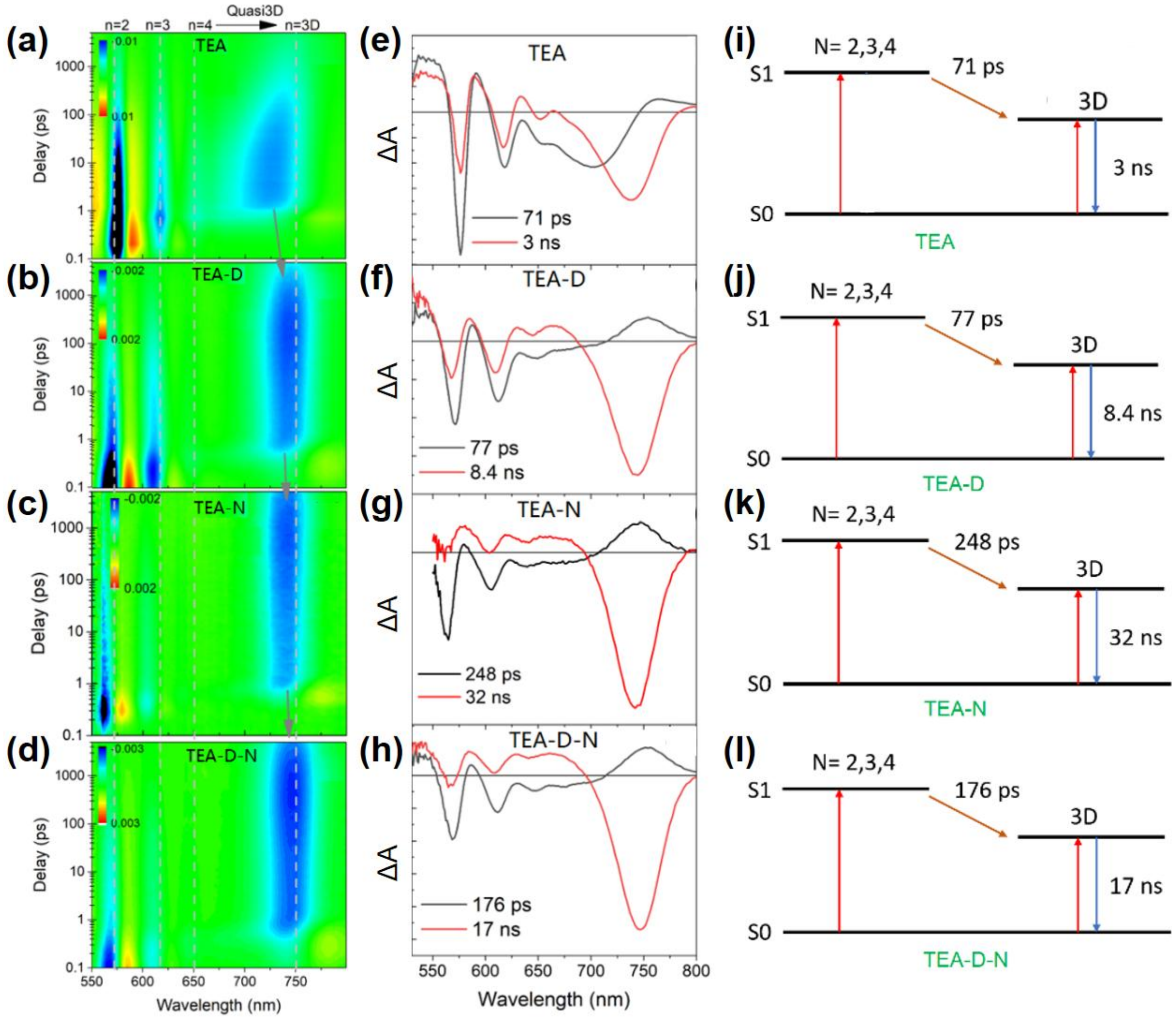

Figure S8. TA spectrograms of (a) TEA, (b) TEA+ DMSO, (c) TEA + NH_4Cl and (d) TEA + NH_4Cl + DMSO excited at 400 nm. The right panel consists of the SVD fitting results (e−h) of all the corresponding TA spectrograms. (i−l) Schematics of charge transfer and recombination pathways of TEA samples analyzed from TA studies.

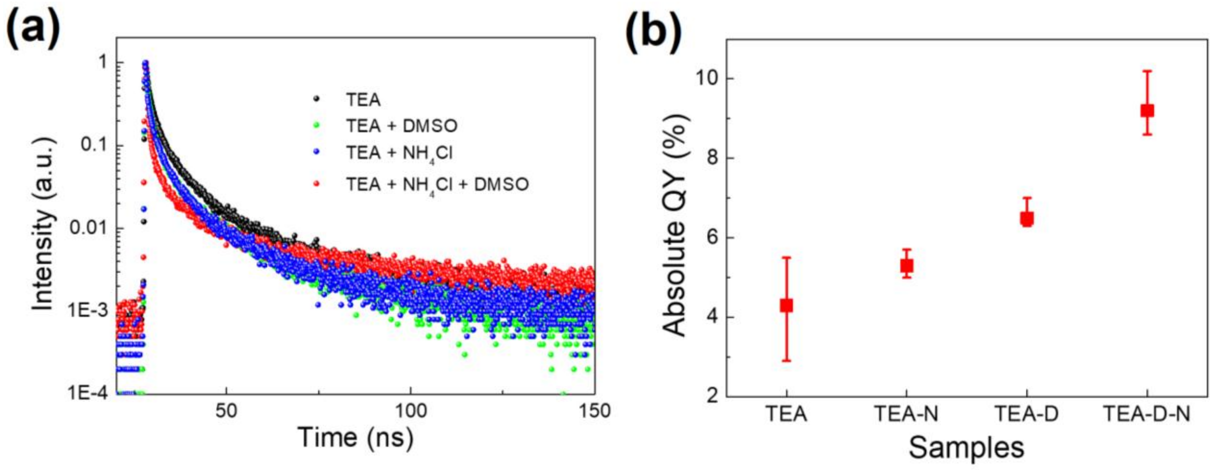

Figure S9. (a) TRPL decays (λ_{ex} = 505 nm) of TEA based 2D perovskites. (b) Absolute PLQY for TEA samples.

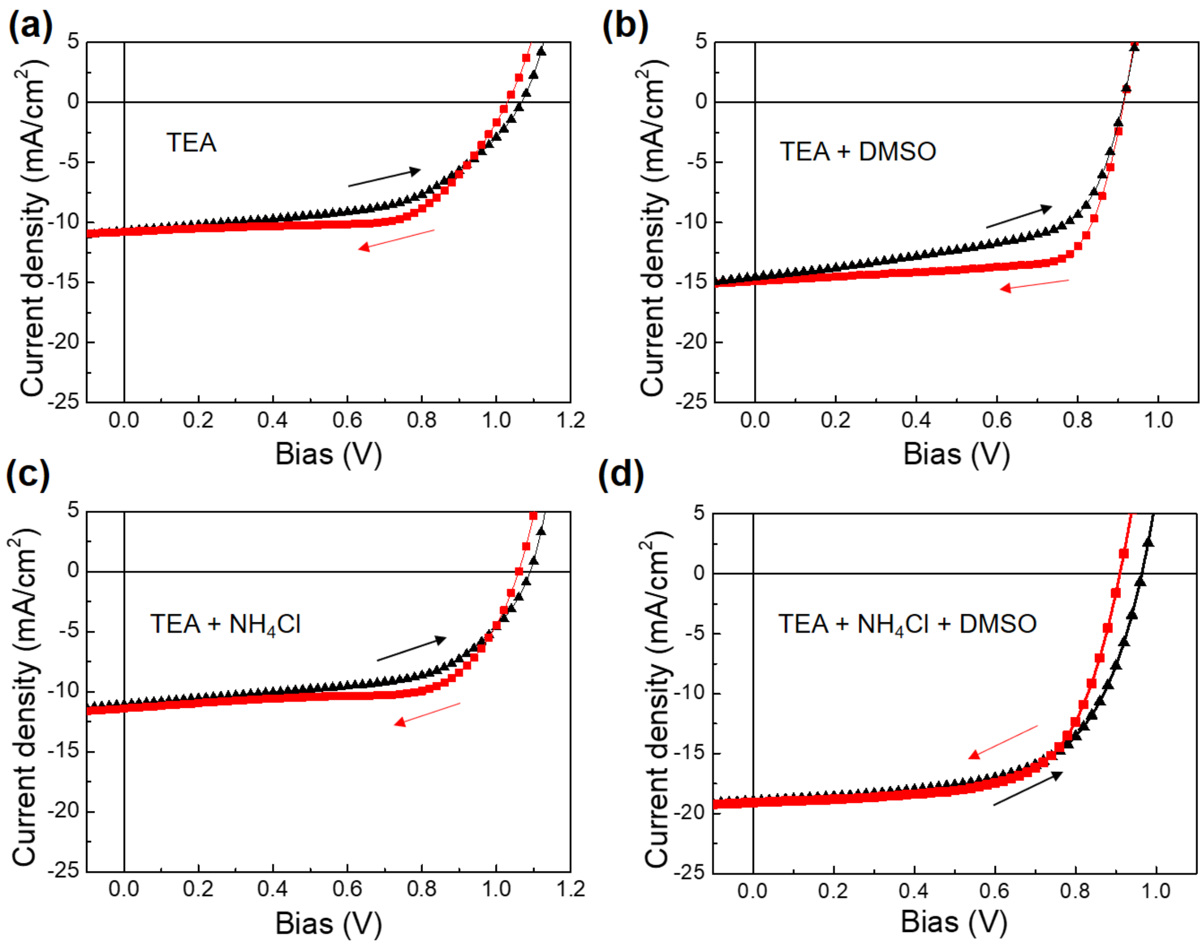

Figure S10. Representative current density–voltage (*J*–*V*) characteristics of devices based on (a) TEA, (b) TEA+ DMSO, (c) TEA + NH_4Cl, and (d) TEA + NH_4Cl + DMSO under AM 1.5G simulated light at forward and reverse scans, respectively.

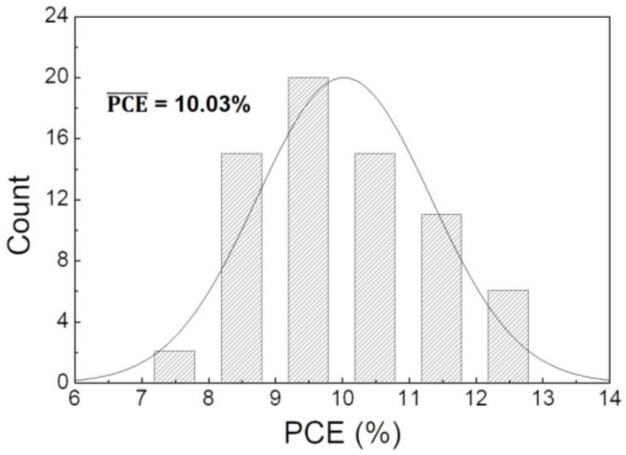

Figure S11. Gaussian distribution of PCEs for TND based PSCs extracted from 69 devices.

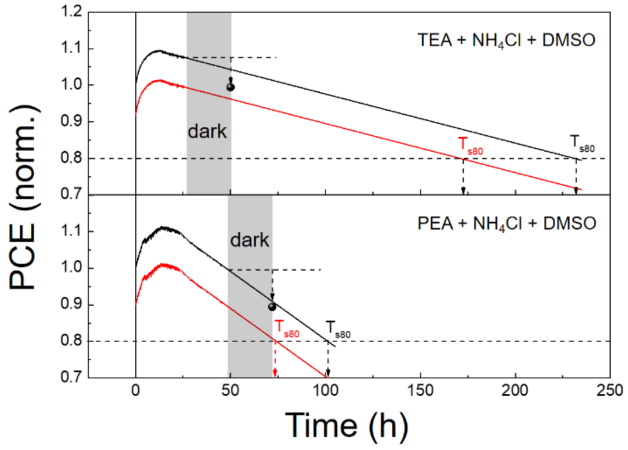

Figure S12. MPP tracking of PND and TND PSCs under a white LED with an intensity of 100 mW/cm^2 at room temperature and a relative humidity of 60 ± 5% before (black) and after (red) dark resting. The devices were kept at open circuit for 24 h in dark and T_{s80} can be extracted from the linear fit of MPP traces after the 'burn-in' section.
